# Attitude, Familiarity and Religious Beliefs about Vaccination among Health Science and Non-Health Science Students in a Malaysian Public University

**DOI:** 10.3390/ejihpe11040104

**Published:** 2021-11-18

**Authors:** Ramdan M. Elkalmi, Eman Dyab, Azyyati Mohd Suhaimi, Ali Qais Blebil, Mohamed Hassan Elnaem, Shazia Jamshed, Márió Gajdács

**Affiliations:** 1 Department of Pharmacology, Faculty of Medicine, University of Sabha, Sabha 00218, Libya; edriph@gmail.com; 2 Department of Pharmaceutics, University of Tripoli, Tripoli 42300, Libya; E.DYAB@uot.edu.ly; 3 Department of Pharmacy Practice, Faculty of Pharmacy, Universiti Teknologi MARA (UiTM), Bandar Puncak Alam, Shah Alam 42300, Malaysia; drazyyati@uitm.edu.my; 4 Clinical Pharmacy, School of Pharmacy, Monash University, Malaysia, Subang Jaya 47500, Malaysia; ali.blebil@monash.edu; 5 Department of Pharmacy, Al Rafidain University College, Baghdad 10001, Iraq; 6 Department of Pharmacy Practice, Kulliyyah of Pharmacy, International Islamic University Malaysia, Kuantan 25200, Malaysia; drmelnaem@iium.edu.my; 7 Department of Clinical Pharmacy and Practice, Faculty of Pharmacy, Universiti Sultan Zainal Abidin, Kuala Terengganu 21300, Malaysia; shaziajamshed@unisza.edu.my; 8 Qualitative Research Group-Pharmacy Practice, Kulliyyah of Pharmacy, International Islamic University Malaysia, Kuantan 25200, Malaysia; 9 Department of Oral Biology and Experimental Dental Research, Faculty of Dentistry, University of Szeged, Tisza Lajos Körút 63, 6720 Szeged, Hungary; 10 Institute of Medical Microbiology, Faculty of Medicine, Semmelweis University, Nagyvárad tér 4., 1089 Budapest, Hungary

**Keywords:** attitudes, knowledge, vaccination, vaccine familiarity, students, religious beliefs

## Abstract

Vaccine hesitancy has surfaced globally within the last few decades, and the fears and misconceptions of people about vaccine safety and effectiveness have been identified as key factors for their under-utilization. The familiarity, attitudes, and religious beliefs of the public and of future healthcare practitioners regarding vaccination are extensive areas needing exploration. The present exploratory cross-sectional study was designed, planned and carried out on students enrolled in health science and non-health science courses in one of the public universities of Malaysia. A research instrument that had been formulated, validated and subjected to reliability testing was used to collect the data, which were analyzed using descriptive and inferential statistics. A response rate of 80.8% (n = 202) was obtained: the majority were female (n = 161, 79.7%), and had been vaccinated before (n = 190, 97.5%), while a mere 2% did not support vaccination for reasons pertaining to safety issues. The vaccine familiarity score was 10.79 ± 1.4, which significantly differed among the study disciplines (*p* < 0.001). The mean of the total attitude score was 14.95 ± 1.5, with no significant difference among demographics being noted. The mean of the total religious beliefs score was 24.29 ± 2.8 and significantly differed based on gender (*p* = 0.040) and study disciplines (*p* < 0.001). The current findings showed that the participants were familiar with vaccines and had generally positive attitudes and positive religious beliefs toward vaccination; thus, one can expect that their inclusion in immunization campaigns will generate positive outcomes of the immunization program. Although the current research reported few knowledge gaps, these may be handled with the introduction of a specialized immunization course at an undergraduate level.

## 1. Introduction

It is undeniable that vaccines—from their first introduction to clinical practice until now—have successfully helped humans to control many specific types of infectious diseases at a reasonable cost, either by eradication or elimination, particularly those that are easily transmitted among children [1,2]. Immunization is recommended by the World Health Organization (WHO) to be used as a preventive measure in both children and adults [1,3]. Immunization is usually achieved by the inoculation of an individual with weakened, killed microorganisms (such as bacteria and viruses) or the toxoid form of these pathogens [4]. This weakened pathogen (or antigen of the pathogen) stimulates the immune system into producing specific mediators, which make the host generate an acquired immunity against this particular pathogen [5]. On a population level, the effectiveness of a vaccination program centers on the number of people who received it [6]. Moreover, its success can be observed by monitoring the number of incidences of that pathogen among the population over a period of time. Although the immunization coverage in developed countries (especially in some Western countries) is better than that in developing ones, a noticeable increase in vaccination coverage in many of these latter countries has occurred after the implementation of a preschool immunization requirement policy [7]. Despite this, an abbreviated adherence toward immunizations has been noted, which could be related to many factors, such as inadequate accessibility, inadequate vaccine supply chains, the compromised availability of health workers, poor motivation on the part of the healthcare staff, lack of resources (logistics), false contraindications, language barriers between caregivers and healthcare workers, negative attitudes and lack of knowledge regarding vaccinations and disease prevention, the uncertainty of vaccination’s effectiveness, fear of adverse events, bearing a female child, and mistrust in the healthcare system [2,6,8,9,10,11,12]. All the abovementioned barriers may lead to the delayed acceptance or complete refusal of vaccination, irrespective of the availability of vaccination services. This “vaccine hesitancy” elicits an upsurge in the proportion of people who do not support vaccination and, therefore, can expose the community to the risk of outbreaks of contagious diseases—previously under control—resulting in the unnecessary suffering of young children and the waste of limited public health resources [2,6,13,14].

The safety of vaccinations has been a matter of concern for parents that has been extensively reported [2,6]. They worry about possible side effects for their children in the future, such as autism spectrum disorder [15], seizures/epilepsy [16], or multiple sclerosis (MS) [17], that have yet to be scientifically proven with any sort of medical evidence [17,18]; however, these may serve as notable fear factors. These factors are leading parents to reconsider vaccination, in turn, boosting the parents’ negative attitudes and perceptions, resulting in unwillingness and disinclination toward the immunization of their own children [7]. Appropriate attitudes, beliefs and familiarity with vaccination are essential factors that lead to the acceptance of vaccination in the population [10]. Vaccines are considered as being safe, but their use may lead to some expected side effects, and some vaccines are not recommended to be used for people with compromised immune systems and during pregnancy [7,10] Live but attenuated viruses or bacteria may cause infections in immunocompromised persons, but they are unlikely to affect individuals with a healthy immune system [19]. However, the benefits of vaccination are far more significant than the risks, according to the conclusions of the American Academy of Pediatrics (AAP), the Centers for Disease Control and Prevention (CDC) and the Institute of Medicine [18,20,21,22].

In Malaysia, the national vaccination program began in 1950; it is a free of charge medical service provided to the public. The program was designed with the aim of preventing and eradicating many contagious communicable diseases, including inoculations for diphtheria, pertussis, tetanus, tuberculosis, poliomyelitis, measles, rubella, mumps and hepatitis. The evidence of the program’s effectiveness can be seen by comparing the number of cases for the particular disease over time in the periods before and after vaccination took place. For example, after the onset of the administration of polio vaccines in 1971, a significant reduction in poliomyelitis prevalence cases has been observed, from 120 cases in 1978 and 20 cases in 1979 to fewer than 5 in 1980 [23]. A similar trend for measles was witnessed, where the number of cases dropped from 300 cases in 1985 to around 130 cases in 1988, after the vaccination coverage increase in 1987. As a result of vaccine strategy implementation, the general population’s health status has improved significantly, especially for the Malaysian population [24], as well as the death rate dropping by approximately 85% from 1970 to 2000 [5,25]. In 2016, the immunization coverage for childhood vaccinations in Malaysia was reported to be within the range of 84.07–99.27% [26]. Most types of vaccines successfully go beyond the optimal level, which is considered to be 95% of the total population [6,8], in order to ensure the success of the “herd immunity” concept [5,7]; however, the Morbilli-Mumps-Rubeola (MMR) and Human Papilloma Virus (HPV) vaccines did not achieve optimum coverage values [26]. Their low coverage was a problem that was observed and reported from 2015 until 2016; it has been attributed to the information spread of anti-vaccine groups or the extensive use of homeopathic therapy [26].

In the current era of COVID-19, any country’s handling of the pandemic strongly relies on how extensively the population agrees to be vaccinated. Findings from countries like Qatar, Italy and United States clearly reported that a large majority of medical, health sciences, and non-health sciences students intended to receive the COVID-19 vaccine, expressed trust in vaccine safety, and expressed trust in public health. These findings are slightly different from data on vaccine hesitancy issues stemming from the general population [27,28,29].

The current research is an attempt to examine the familiarity with the process of vaccination, evaluate attitudes toward vaccines, and explore the religious beliefs of health science and non-health science students regarding vaccination in one of the public universities in Malaysia.

## 2. Materials and Methods

### 2.1. Study Design

The present study is of a descriptive, exploratory, and cross-sectional design, employing a questionnaire as a data collection instrument; the research was conducted from March to June 2019 at the Universiti Teknologi Mara (UiTM)—Puncak Alam Campus (Selangor, Malaysia).

### 2.2. Study Population and Sampling Method

The targeted study population was final-year students attending different faculties of the UiTM, including the Faculty of Pharmacy and Faculty of Nursing, to represent health science students. In contrast, non-health science students were exemplified by students from the Faculty of Accounting and the Faculty of Art and Design. The sampling frame consisted of all full-time students, who enrolled in their fourth year at their respective faculties, as mentioned above, during the study period; the full number of students was obtained from the respective Deanship in each Faculty (n = 460). The sample size was determined proportionally, based on the sampling frame; the calculated minimum sample size was n = 210, considering a 20% excess for dropouts or non-responders, with a confidence interval of ±5% and a confidence coefficient of 95%. In all, a total sample size of 250 was obtained [30].

### 2.3. Study Instrument

The initial draft of the questionnaire was constructed and developed, based on previously published studies regarding vaccination and religion-related issues [1,3,7,30,31,32,33,34]. Three pharmacy lecturers, with experience in vaccination and social pharmacy studies, were asked to evaluate the relevancy, clarity, and conciseness of the items included in the questionnaire. Their observations and comments were taken into consideration to create the final version of the questionnaire. In order to test the reliability of the survey form, the revised questionnaire was pilot-tested by administering it to a sample of 5 students from each individual faculty. Those participants were excluded from the main study. The reliability of the questionnaire was assessed by calculating Cronbach’s alpha reliability coefficient; the α value calculated for our instrument was 0.78, which corresponds to good internal consistency. The resulting final version of the questionnaire was composed of 38 items, distributed over five sections, including demographic information, reasons for not supporting vaccination, general attitudes toward vaccination, religious beliefs toward vaccination, familiarity regarding vaccinations, and students’ religious activities. However, the survey results related to each respondent’s religious activities were excluded from the analysis and from being detailed in this article.

### 2.4. Study Procedure

The objectives of the survey were explained to the students through an explanatory letter attached to the survey questionnaire that was distributed to all participants. The students received the survey questionnaire through the respective lecture coordinators at each faculty. Anonymity and confidentiality were guaranteed. Consent for participation was implied by the completion and return of the survey instrument. The UiTM Research Ethics Committee gave their approval (REC/239/19).

### 2.5. Statistical Analysis

All data obtained from the survey were analyzed using the International Business Machine statistical package for the social sciences v. 21 (IBM SPSS Inc., Chicago, IL, USA). Descriptive and inferential statistics were applied; categorical data are presented as percentages and frequencies. Any associations or differences between groups were examined by the chi-square or Fisher exact tests, as appropriate. When appropriate, Student’s *t*-tests were performed by comparing the means of two continuous variables. A one-way ANOVA with a post hoc Tukey HSD (honestly significant difference) has been used for multiple comparisons, in order to detect the existence of differences between pairwise groups. All statistical tests were two-tailed and maintained a significance level (α) < 0.05 and a confidence interval (CI) > 95%.

## 3. Results

A final sample of n = 250 students was recruited for the study. Only 202 answered the questionnaire, generating a response rate of 80.8%. Respondents were in the age range of 21 to 24 years (mean 22.8 ± 0.62 years). The majority of them were female (n = 161, 79.7%), while almost all of the study participants indicated that they had previously been vaccinated (n = 190, 97.5%). In all, only four (2%) students did not support the vaccination idea at all, and their main reason for this attitude was related to the safety issues that could be associated with the vaccines (4.2%). More details about the sociodemographic variables and the identified reasons for not supporting immunization are presented in Table 1 and Table 2, respectively.

The total mean familiarity score for the respondents in the whole study was 10.787 ± 1.4 (range 7.0–14). There was a statistically significant difference in the mean score of familiarity with vaccination in terms of the field of study (health science vs. non-health science; *p* < 0.001). Additionally, a statistically significant difference in familiarity with vaccination concerning the current study course of the respondents has also been noted (*p* < 0.001). Post hoc analysis (Tukey HSD) of the data showed that students from the Faculty of Art and Design exhibited knowledge, compared to students from the Faculties of Pharmacy and Nursing (*p* < 0.001, *p* < 0.001, respectively). In addition, students from the Faculty of Accounting were more familiar with vaccination than students from the Faculties of Pharmacy and Nursing (*p* < 0.001, *p* < 0.001, respectively). No statistically significant difference was seen between the students of the Faculties of Pharmacy and Nursing regarding the total score of their familiarity with vaccination (*p* = 0.646). No relevant differences were seen in the mean score of familiarity with vaccination, with regard to gender, vaccination status and the supporting attitude toward vaccination (*p* = 0.448, *p* = 0.739 and *p* = 0.200, respectively). The mean scores of familiarity with vaccination among the study respondents, tabulated according to their demographic characteristics, are shown in Table 3.

Study findings showed that almost all (n = 201, 99.5%) of the study respondents demonstrated their familiarity with vaccination. Indeed, comparatively few of them affirmed their lack of understanding of how vaccines work (n = 34, 16.8%), while the majority seemed sure about it (n = 168, 83.2%). There was a significant difference in responses to this statement with regard to gender, field of study, vaccination status and underlying attitude toward vaccination (*p* = 0.007 (female), *p* < 0.001 (health sciences), *p* = 0.003 (health sciences) and 0.016 (female), respectively). The study findings showed that three-quarters (n = 153, 75.7%) of respondents showed their lack of knowledge of the National Immunization Awareness Month (NIAM) in Malaysia. A significant statistical difference was observed among students of different fields of study, with regard to the familiarity with the NIAM. However, the vast majority of respondents (n = 87, 88.8%) who demonstrated a lack of familiarity with the NIAM were from the non-health science field. Only a tiny percentage (n = 12, 5.9%) of the study respondents agreed with the statement that vaccination leads to autism (Table 4).

Overall, study respondents had positive attitude scores regarding vaccination (14.946 ± 1.5). There was no significant difference in mean attitude scores (*p* = 0.890) between males (14.967 ± 1.7) and females (17.938 ± 1.5), between study fields (health science/non-health science) (*p* = 0.549) and among the different faculties (*p* = 0.098). Despite that, study findings indicated that accounting (15.127 ± 1.9), pharmacy (15.039 ± 1.2) and nursing students (15.038 ± 1.0) had predictably higher (more positive) attitude scores than did art and design students (14.361 ± 0.2) (Table 3).

The vast majority (n = 181, 89.6%) of the respondents indicated that they were being vaccinated because they believed in the safety of vaccines. There was a significant difference in response to this statement among the various study field groups (*p* = 0.003). Almost all of the respondents who agreed with this statement (n = 100, 96.2%) were from the health science field. Slightly over two-thirds (n = 153, 75.7%) of respondents in the study expressed that they intend to recommend vaccines to the people around them. There was a significant difference (*p* < 0.001) in response to this statement between students among the various fields of study. The overwhelming majority (n = 58, 59.2%) of non-health science students agreed with this statement. Fewer than forty percent (n = 79, 39.1%) of students agreed that the Ministry of Education should reject the enrolment of unvaccinated children into public schools. There was no association between this statement and gender, field of study or vaccination status (*p* = 0.575, *p* = 0.111, *p* = 1 and *p* = 1, respectively) (Table 5).

Overall, our respondents had positive religious belief scores toward vaccination (24.287 ± 2.8). There was a statistically significant difference between religious belief scores, based on gender (males vs. females; 23.463 ± 3.5 vs. 24.497 ± 2.6; *p* = 0.04), the field of study (health science/non-health science) (*p* < 0.001), the current study course (*p* < 0.001) and the different faculties (*p* < 0.001). Post-hoc comparisons using the Tukey HSD test indicated that pharmacy students (25.052 ± 2.0) and nursing students (24.731 ± 1.8) had significantly more positive religious belief scores than accounting students (24.667 ± 3.4) and art and design students (21.667 ± 5.2) (Table 6).

Almost all (n = 183, 90.6%) of the participants believed that they should have the vaccine to maintain health because the body is an *Amanah* (trust) from God (Allah). There was a significant association in response to this statement with vaccination status and vaccination support (*p* = 0.008 and *p* = 0.020, respectively). Almost all (n = 180, 91.4%) of the respondents who agreed to this statement were those who had been vaccinated before and who (n = 180, 90.9%) supported vaccination. More than half (n = 121, 59.9%) of respondents to this study believe that JAKIM must issue a fatwah that makes the vaccination of children obligatory for parents. There was a significant association between responses to this statement and the students’ study field (*p* = 0.020). Our results indicate that most respondents who supported this statement are health-science students (n = 70, 67.7%) compared to non-health science students (n = 51, 52.0%). Nearly half of the study participants believed that vaccination may cause some side effects. A statistically significant association was observed between the response to this statement and the field of study, vaccination status, and the support of vaccination (*p* = 0.000, *p* = 0.042 and 0.035, respectively). It was noted that the vast majority (n = 71, 68.3%) of medical field students believed that vaccination might cause side effects in some people, compared to half of the non-medical field students who were neutral (n = 49, 50.0%) (Table 6).

## 4. Discussion

To the best of our knowledge, only a few studies have been carried out in Malaysia, addressing the attitudes, familiarity, and religious beliefs of university students toward vaccination [3,32,33]. In our study, most study participants showed positive attitudes toward vaccination; in general, students in the field of health sciences had significantly more positive attitude scores than students in the field of non-health sciences. This variation could be attributed and explained logically since medical-field students are more exposed to basic information about epidemiology and the microbiological and immunological bases of vaccination. The findings indicating a positive attitude in undergraduate students toward vaccinations, which is similar to the results reported for their peers in other universities and in healthcare professionals in previous studies [3,8,32,33,34,35], bearing in mind that the current research findings may not be confidently extrapolated to other undergraduate students in other universities. It would be beneficial to emphasize the importance of vaccination to undergraduate students and encourage their involvement in advocating vaccination, not only to their colleagues but also to friends, family members and the general public [36].

Overall, respondents in the current research exhibited a comforting familiarity with vaccination. However, the majority expressed a lack of familiarity with the National Immunization Promotion Campaign 2016–2020 (NIPC 2016–2020). The NIPC is an initiative, led by the Ministry of Health of Malaysia, to address the issue of vaccine rejection in the community and the strengthening of the national immunization program in the country [26]. The NIPC initiative aims to provide the Immunization Kit helping in the education and training of vaccine advocates among family health specialists/doctors/paramedics, as well as organizing and participating in the forums and seminars conducted by states in collaboration with other health agencies at the local level.

Interestingly, non-medical students showed better familiarity with vaccination as compared to medical students. Further investigation to explore this variation is highly recommended. The current findings are inconsistent with the previous study, wherein medical students had higher knowledge scores than non-medical students [37]. Our findings also contradict the results of a similar Turkish study that showed a more pronounced lack of vaccination-related knowledge among the non-medical students compared to their medical counterparts [38]. On the other hand, relatively low levels of knowledge regarding vaccination among our medical participants are consistent with the findings of a Chinese study that highlighted a low level of knowledge in medical students regarding HPV vaccination, where almost all study participants revealed that they really do not understand how these vaccines work [39]. Similarly, previous research highlighted that a lack of knowledge regarding the recommended vaccinations constitutes a universal challenge in vaccination uptake among adolescents [16]. The study failed to show any impact of the vaccination status among study respondents on their familiarity level with vaccination; this might be attributed to the sample size, where very few respondents showed their lack of support for vaccination.

In relation to the previous results, only the most frequently reported reason to hesitate and/or refuse vaccinations was identified—the fear of side effects, referred to by all subjects who self-reported as being against vaccinations, followed by the fear of injections (50.0%), along with a series of statements such as the lack of trust in vaccines, or the belief that immunization by natural immunity would be more efficient than that promoted by vaccination. Several reasons might contribute to non-vaccination, such as parental attitudes and knowledge, immunization systems, and family characteristics [12]. In the current study, although few participants declared themselves as being against the practice of vaccination—they were able to disclose their reasons behind their anti-vaccination attitude, such as the frequently mentioned risk, the lack of trust in vaccines, and the belief that there is no need for external methods to boost the immune response of the body. Parental knowledge and attitudes were thought to be affected by religious beliefs, where many unvaccinated or under-vaccinated groups were found to be religion-based ones [13].

In our study, we found a difference in the religious beliefs, based on study fields where the medical participants have demonstrated more positive religious attitudes toward vaccination. This might be explained through changes in the mindset of those involved in the medical field, arguing that interventions such as vaccination should be evaluated according to the overall potential benefits while understanding that no intervention is free from risks. Moreover, our findings have underpinned gender-based differences in the participants’ beliefs regarding vaccination. In contrast, a similar study conducted among medical and non-medical students did not show any differences in the attitudes of participants based on their gender [38].

The importance of receiving vaccinations in the prevention of contracting infectious ailments and in the reduction of antimicrobial resistance (by limiting the prescribing of drugs) cannot be underestimated [40,41]. It is pertinent to evaluate the depth of the preventive health content of the curriculum and, if required, training programs for the trainers will be instituted to strengthen their capabilities [42]. As highlighted and introduced by Louizos et al., there is a global need to initiate a similar sort of pharmacy immunization and injection training program through interprofessional collaboration, to train pharmacists and other future healthcare practitioners in the facilitation of immunization [43]. Last but not least, vaccination programs will be recommended to undergraduate students in non-health sciences disciplines; they should be offered vaccine choices like inactivated vaccines and live attenuated vaccines for influenza prevention and encouraged to promote immunization through leaflets, online information, and healthcare providers [44].

Firstly, as this is cross-sectional research that is focused on only one university, the findings are not generalized to other university students. Secondly, the instrument is not subjected to factor analysis; only the instrument was validated by subjecting it to the scrutiny of an expert panel.

## 5. Conclusions

The current findings reported a positive attitude in students toward vaccination activities in Malaysia. These significant findings reflect the possibility of including the students in vaccine promotion and neutralizing false perceptions about immunization.

In terms of addressing the knowledge gaps among the study participants, comprehensive, specialized, educational immunization programs are recommended for undergraduate students. The outcomes of this research may highlight the necessity for targeted interventions to overcome the issue of low vaccine coverage by modifying knowledge and attitudes, helping to prepare future parents and healthcare workers for playing an important role in immunization plans. Future healthcare practitioners can be instrumental in strengthening and disseminating knowledge by informing through evidence-based practices, addressing concerns about vaccine uptake and safety in their social communities. Likewise, policymakers can accelerate their efforts and strengthen the lay public’s decision-making around immunization by promoting a sense of community and effectively addressing conspiracy theories.

## Figures and Tables

**Table 1:** Demographic characteristics of respondents (n = 202).

Demographics	Frequency (n)	(%)
**Age (mean ± SD, 22.8 ± 0.62)**		
21	3	(1.5%)
22	55	(27.2%)
23	125	(61.9%)
24	19	(9.4%)
**Gender**		
Male	41	(20.3%)
Female	161	(79.7%)
**Marital status**		
Single	200	(99%)
Married	2	(1%)
**Respondent’s courses**		
Pharmacy	77	(38.1%)
Nursing	26	(12.9%)
Accountancy	63	(31.2%)
Art and design	36	(17.8%)
**Respondent’s group**		
Health science students	104	(51.5%)
Non-health science students	98	(48.5%)
**Have you ever been vaccinated before?**		
Yes	190	(97.5%)
No	5	(2.5%)
**Do you support vaccination?**		
Yes	198	(98.0%)
No	4	(2.0%)

**Table 2:** Reasons for not supporting vaccination among non-supporters (n = 4).

Reasons	Agree	Neutral	Disagree	Total
	f (%)	f (%)	f (%)	f (%)
Prohibited in Islam	3 (1.5)	1 (0.5)	0	4 (100)
Halal issue of vaccine	3 (1.5)	1 (0.5)	0	4 (100)
Prohibited by my family	3 (1.5)	0	1 (0.5)	4 (100)
Risks associated with vaccination	4 (2.0)	0	0	4 (100)
Considers it an immoral activity	3 (1.5)	1 (1.5)	0	4 (100)
The negative view of the public	3 (1.5)	0	1 (0.5)	4 (100)

**Table 3:** Variations in total familiarity and attitude score among study participants, along with their sociodemographic characteristics (n = 202).

Sociodemographic Character	Familiarity		Attitudes		Religious Beliefs	
Details (*f*)	Mean (SD)	*p*-Value ^a^	Mean (SD)	*p*-Value ^a^	Mean (SD)	*p*-Value ^a^
Gender						
Male (41)	10.634(1.2)	0.448	14.976(1.7)	0.890	23.463(3.5)	**0.040**
Female (161)	10.824(1.4)		17.938(1.5)		24.497(2.6)	
Study field						
Medical (104)	9.8849(0.8)	**0.001**	15.010(1.2)	0.549	24.981(1.9)	**0.000**
Nonmedical (98)	11.745(1.3)		14.848(1.8)		23.551(3.4)	
Vaccination status						
Yes (197)	10.782(1.4)	0.739	14.970(1.4)	0.170	24.345(2.6)	0.072
No (5)	11.000(1.2)		14.000(4.2)		22.000(8.0)	
Did you support vaccination?						
Yes (198)	10.803(1.4)	0.200	14.985(1.4)	**0.011**	24.323(2.6)	0.211
No (4)	10.000(1.8)		13.000(4.1)		22.500(9.2)	
Study Course						
Pharmacy (77)	9.818(0.7)	**0.001** ^b^	15.039(1.2)	0.098 ^b^	25.052(2.0)	**0.001** ^b^
Nursing (26)	10.115(1.1)		15.038(1.0)		24.731(1.8)	
Accounting (63)	11.683(0.1)		15.127(1.9)		24.667(3.4)	
Art and Design (36)	11.778(0.2)		14.361(0.2)		21.667(2.5)	

^a^ Independent sample *t*-test. ^b^ One-way ANOVA with post hoc Tukey HSD; *p* values in **boldface** represent statistically significant differences.

**Table 4:** Familiarity of participants with vaccination in relation to their sociodemographic information (n = 202).

Questions/Statements	Responses		*p*-Value			
	Yes (n, %)	No (n, %)	Gender	Study Field	Vaccination Status	Vaccination Support
Have you ever heard about vaccination?	201 (99.5)	1.0 (0.5)	1.00	0.485	1.000	0.887
I don’t really understand how vaccines work.	34 (16.8)	168 (83.2)	**0.007**	**0.001** W > M	**0.003** W > M	**0.016** W > M
Vaccination is the transfer of weakened microbes into the human body to generate antibodies, to protect against a particular disease.	112 (55.4)	90 (44.6)	0.726	**0.001**	0.174	1.000
There is a National Immunization Awareness Month (NIAM) organized in Malaysia.	49 (24.3)	153 (75.7)	0.839	**0.001**	0.339	1.000
Vaccines have succeeded in reducing many infectious diseases.	123 (60.9)	79 (39.1)	0.858	**0.001**	0.078	1.000
It is possible for someone to have many types of vaccination.	118 (58.4)	84 (41.6)	0.595	**0.001**	0.163	1.000
Vaccination leads to autism.	12 (5.9)	190 (94.1)	**0.070**	0.560	**0.002** W > M	**0.018** W > M

W = Women; M = Men; *p* values in **boldface** represent statistically significant differences.

**Table 5:** Attitude of participants toward vaccination in relation to their sociodemographic information.

Statements	Responses			*p*-Value			
	Agree f (%)	Neutral f (%)	Disagree f (%)	Gender	Study Field	Vaccinating Status	Vaccination Support
I am taking the vaccines because they are safe for me.	181 (89.6)	20 (9.9)	1 (0.5)	0.105	**0.003**	**0.009** P > N	**0.005** P > N
I am taking the vaccines because they are easily accessible.	133 (65.8)	67 (33.2)	2 (1.0)	0.734	0.365	1.000	0.619
I trust pharmaceutical companies to provide safe and effective vaccines.	150 (74.3)	51 (25)	1 (0.5)	0.144	**0.043**	**0.025** P > N	**0.020** P > N
I recommend vaccination for friends and family around me.	153 (75.7)	47 (23.3)	2 (1.0)	0.247	**0.001**	**0.012** P > N	**0.006** P > N
The use of alternative practices, such as homeopathy, can replace vaccination.	27 (13.4)	96 (47.5)	79 (39.1)	0.676	**0.001**	0.268	1.000
The Ministry of Education should reject the enrolment of unvaccinated children into public schools.	79 (39.1)	80 (39.6)	39 (21.3)	0.575	0.111	1.000	1.000

P = Pharmacy; N = Nursing; *p* values in **boldface** represent statistically significant differences

**Table 6:** The religious belief of participants toward vaccination in relation to their sociodemographic information.

Questions I Believe That…	Responses			*p*-Value			
	Agree (%)	Neutral (%)	Disagree (%)	Gender	Study Filed	Vaccinating Status	Vaccination Support
I take the vaccines to maintain my health because my body is the *Amanah* from Allah that I must take care of.	183 (90.6)	18 (8.9)	1.0 (0.5)	0.099	0.270	**0.008** P > N	**0.020** P > N
In Islam, the need for vaccination is parallel with the law to protect life and the principle of preventing harm (*izalat aldharar*).	176 (87.1)	25 (12.4)	1 (0.5)	0.168	**0.001**	**0.010** P > N	**0.020** P > N
I take the vaccine even though it is porcine-based, but with justification from *maqasid syariah*.	86 (42.6)	75 (37.1)	41 (20.3)	**0.002**	**0.013**	0.637	0.196
*Sunnah* food, such as honey, can replace vaccination in promoting health.	28 (13.9)	99(49.0)	75 (37.1)	1.000	**0.001**	0.273	0.070
Vaccination is an effort (*ikhtiar*) to acquire a better quality of life.	168(83.2)	31(15.3)	3 (1.5)	0.186	**0.001**	0.075	**0.024** P > N
Religion becomes a barrier to vaccination.	36 (17.8)	50 (24.8)	116 (57.4)	0.741	0.493	0.292	0.140
Vaccination is *halal*.	111 (55.0)	86 (42.6)	5 (2.5)	0.082	**0.009**	0.077	0.096
Vaccination is consistent with Islamic principles.	131 (64.9)	70 (34.7)	1 (0.5)	0.129	**0.000**	**0.015** P > N	**0.020** P > N
*JAKIM* has to deliver a *Fatwa* that makes the vaccination of children obligatory for parents.	121 (59.9)	71 (35.1)	10 (5.0)	0.542	0.063	**0.020** P > N	0.200
A vaccine may cause side effects in certain people.	102 (20.5)	77 (38.1)	23 (11.4)	0.138	**0.000**	**0.042** P > N	**0.035** P > N

*JAKIM: Jabatan Kemajuan Islam Malaysia,* Islamic Development of Malaysia; *Fatwa*: a ruling on a point of Islamic law given by a recognized authority. P = Pharmacy; N = Nursing; *p* values in **boldface** represent statistically significant differences.

## Data Availability

All data generated during the study are presented in this paper.
